# Passing the post: roles of posttranslational modifications in the form and function of extracellular matrix

**DOI:** 10.1152/ajpcell.00054.2023

**Published:** 2023-03-13

**Authors:** Josephine C. Adams

**Affiliations:** School of Biochemistry, University of Bristol, Bristol, United Kingdom

**Keywords:** breast cancer, cell adhesion, extracellular matrix, secretome

## Abstract

The extracellular matrix (ECM) is central to the physiology of animal tissues, through its multifaceted roles in tissue structure, mechanical properties, and cell interactions, and by its cell-signaling activities that regulate cell phenotype and behavior. The secretion of ECM proteins typically involves multiple transport and processing steps within the endoplasmic reticulum and the subsequent compartments of the secretory pathway. Many ECM proteins are substituted with various posttranslational modifications (PTMs) and there is increasing evidence of how PTM additions are required for ECM protein secretion or functionality within the extracellular milieu. The targeting of PTM-addition steps may thus offer opportunities to manipulate ECM quality or quantity, in vitro or in vivo. This review discusses selected examples of PTMs of ECM proteins for which the PTM has known importance for anterograde trafficking and secretion of the core protein, and/or loss-of-function of the respectively modifying enzyme leads to alterations of ECM structure or function with pathophysiological consequences in humans. Members of the protein disulfide isomerase (PDI) family have central roles in disulfide bond formation and isomerization within the endoplasmic reticulum, and are discussed in relation to emerging knowledge of the roles of certain PDIs in ECM production in the pathophysiological context of breast cancer. Cumulative data suggest the possible applicability of inhibition of PDIA3 activity to modulate ECM composition and functionality within the tumor microenvironment.

## INTRODUCTION

The interactions of metazoan (animal) cells with extracellular matrix (ECM) and their physiological and pathological roles have interested me throughout my career. These interactions arise from the complex relationship, sometimes termed “dynamic reciprocity,” in which cells are both the producers of ECM and also responders to mechanical and chemical cues from the ECM which control cell phenotypes and cell behavior. Metazoan cells do not have a cell wall and so their interactions with ECM proteins are mediated directly by plasma membrane-located receptors such as integrins or syndecan proteoglycans. These communications, in concert with intracellular pathways of cell regulation or other categories of extracellular mediators, determine the form, mechanical properties, and functions of tissues and organs (1, 2). Given that ECM proteins are secreted by cells, research in this field inherently considers the lifecycle of proteins between the intracellular and extracellular compartments and the role of the plasma membrane in between. Hugh Davson’s model with James Danielli of plasma membrane structure, the Davson–Danielli model, informed thinking about membrane properties and the workings of cells for decades until the arrival of the fluid mosaic model proposed by Singer and Nicholson (3, 4). Subsequently, Hugh Davson worked extensively on the extracellular milieu of tissues in the form of the ocular and cerebrospinal fluids (5). My lecture took the centrality of the plasma membrane as a starting point from which to discuss ECM-related research which has emphasized connections and communications between the extracellular and intracellular milieus. This review develops one area in which my laboratory has become involved in recent years: the roles of posttranslational modifications (PTMs) of ECM proteins in ensuring their trafficking, secretion, and functionality within ECM.

It is estimated that around 11% of the proteins encoded in the human genome are destined for secretion. These polypeptides, along with the transmembrane proteins, enter the lumen of the rough endoplasmic reticulum (ER) cotranslationally to undergo folding, quality control, and anterograde transport to the final site of function. Chaperones or cargo-sorting proteins have essential roles in onward trafficking of correctly folded ECM proteins, one example being the sorting of procollagen into COPII vesicles by SEC24 paralogues (6). In traversing the ER and Golgi apparatus, ECM proteins also undergo an array of posttranslational modifications. This topic represents a huge area of cell physiology: around ∼300 ECM-protein coding sequences can be identified in the human and mouse genomes by in silico methods (7) and proteomic studies of ECM isolated from cultured mammalian cells or dissected tissues (the “matrisome”) has expanded the total number of possible matrisomal components to over 1,000 (8, 9). The majority of these proteins have undergone multiple forms of PTM, ranging from disulfide bonds necessary for the structure of individual domains or oligomerization of polypeptides, to many forms of N- or O-linked glycosylation, phosphorylation, hydroxylation, or acetylation, among others. The complexity of the in vivo situation is illustrated by mass spectrometry methods that facilitate the detection of PTMs. A study of decellularized (i.e., ECM-enriched) preparations of human pancreatic tissue undertook electrostatic repulsion-hydrophilic interaction chromatography to enrich for N-glycosylated and phospho-peptides, followed by mass spectrometry proteomics. Of 214 identified matrisomal proteins, N-glycosylation was detected on 99 proteins, phosphorylation was detected on 18 proteins including several collagen chains, and 9 proteins, including collagen VA1, carried both PTMs (10). In a study of mouse mammary tumors involving a photocleavable surfactant and the solubilization of ECM for mass spectrometry proteomics, 225 unique ECM proteins were identified carrying a total of 229 PTMs that included glycosylation, phosphorylation, and hydroxylation (11).

With regard to this vast scope of molecular heterogeneity, this review does not aim to provide an exhaustive survey but will discuss selected examples of PTMs on specific ECM proteins for which the PTM has known importance for protein production, or its absence leads to clear pathophysiological consequences in humans. This is of interest because manipulation of specific PTM-addition steps may offer new therapeutic possibilities to inhibit or correct localized pathological alterations to the ECM, such as occur in cancer or tissue fibrosis. I will then focus on members of the protein disulfide isomerase (PDI) family, that have central roles in disulfide bond formation and isomerization within the ER, to discuss emerging knowledge of the significance of certain members of this family in the pathophysiological context of breast cancer. Emerging data suggest the possible applicability of PDIA3 inhibition to modulate ECM composition and functionality in invasive breast cancer.

## PTMs: ROLES IN THE STRUCTURE AND FUNCTION OF ECM PROTEINS

### O-Fucosylation of Thrombospondin Type 1 Domains: Roles in Protein Secretion

The forward trafficking of newly synthesized ECM proteins from the ER relies not only on sorting cues for carriage in the correct trafficking vesicles but also on the addition of PTMs to the core protein that may be essential for its further processing, maturation and/or final structure, or functionality. Without these additions, the protein in question is typically recognized as misfolded and either undergoes retrograde transport for further cycles of folding, or may be targeted for destruction by ER-associated degradation, involving the unfolded protein response pathway and delivery to the proteasome or lysosome (12). Such failures of secretion also have downstream consequences for ECM composition, organization, and functional properties. An example of the importance of individual PTMs and the existence of interrelationships between different forms of PTM at the domain level is provided by the thrombospondin type 1 domain (TSR). The TSR is a common domain present as tandem repeats in ECM glycoproteins of the thrombospondin family, a variety of other ECM and cell-surface associated proteins and in members of the “a disintegrin and metalloproteinase with thrombospondin motifs” (ADAMTS) family of extracellular proteases which act on many ECM protein substrates (13).

**Figure 1.**
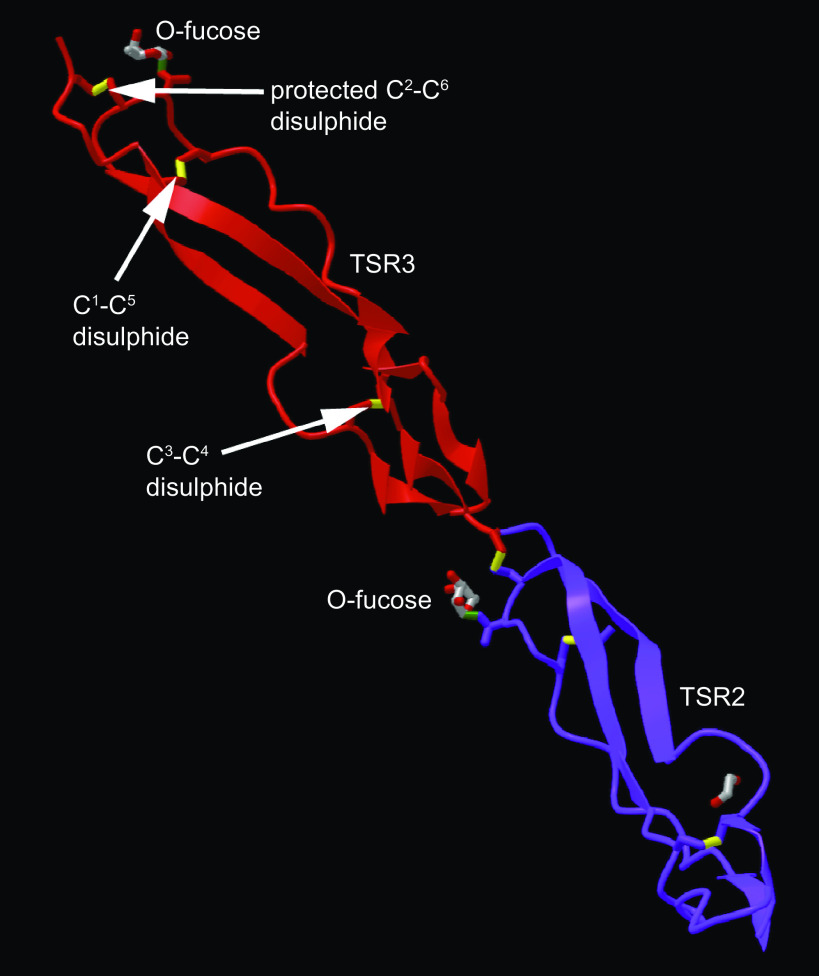
Posttranslational modifications support stable folding of the TSR domains of thrombospondin-1. The structural model [PDB 7YYK (14)] shows the second (purple) and third (red) TSR domains. Disulfide bonds are in yellow, with cysteines numbered from the N-terminus of the domain. The position of the O-fucose modification that shields a disulfide bond to stabilize the folded domain is indicated in TSR3. Diagram prepared in iCn3D (15) and exported from NCBI. TSR, thrombospondin type 1 domain.

TSRs typically function in protein-protein or protein-glycosaminoglycan interactions and are small (∼60 amino acids) domains, which contain six cysteine residues that form three disulfide bonds (each formed by oxidation of the thiol groups of two cysteine resides; Fig. 1). These bonds confer the characteristic structural fold of the TSR (16). Folded TSR that contain the motif Cys^1^-X-X-(Ser/Thr)-Cys^2^ or Cys^2^-X-X-(Ser/Thr)-Cys^3^ (numbers refer to the numbering of the Cys residues within a TSR) can then be further modified by O-linkage of fucose onto the hydroxyl group of the serine or threonine residue within the motif. This reaction is catalyzed by the ER enzyme Protein O-fucosyltransferase 2 (POFUT2; 17). Addition of glucose by β3-glucosyltransferase (B3GLCT) then assembles a characteristic Glucoseβ1–3Fucose disaccharide (18). In humans, O-fucosylation consensus motifs are present in the TSR of around 50 proteins, including many ADAMTS proteins. A number of these proteins have been found to depend on O-fucosylation of TSR for their secretion (19), through a proposed stabilizing effect of the sugar modification on the folded TSR structure (20). The structural basis of stabilization has been studied in the context of the TSR3 domain of thrombospondin-1. Contacts between the disaccharide and side groups of underlying amino acids orientate the sugar such that it protects the Cys^2^ to Cys^6^ disulfide bond from reduction within the ER (Fig. 1). This can be interpreted as a quality control mechanism that maintains the folded, disulfide-bonded state of target TSRs, promoting onward trafficking and secretion of the respective protein (14).

The general physiological importance of the O-fucose-based disaccharide as a PTM is evident from the effects of mutations in *B3GLCT* that lead to loss of function of the catalytic domain and cause the human genetic disorder Peters plus syndrome, characterized by ocular and craniofacial defects, short stature, and a spectrum of other developmental delays (OMIM 261540). Various disease-causing mutations of *B3GLCT* reduce its enzymatic activity or protein stability (21). Whereas gene-knockout of *pofut2* in mice is early embryonic lethal during gastrulation (22), the phenotypes of *b3glct*-null mice broadly mimic those of the human Peters plus syndrome (23). The phenotypic effects of knockout of various *adamts* genes in mice also overlap with the clinical phenotypes caused by *B3GLCT* mutations in humans, indicating essential physiological roles for O-fucosylation of the many TSRs of ADAMTS proteins (24). For example, O-fucosylation of TSRs in ADAMTSL2 was found to be essential for secretion and thereby its actions on its extracellular substrates (25).

The activity of POFUT2 and other fucosyl transferases can be monitored or inhibited through the use of fucose analogs. Synthetic fucose derivatives designed for click chemistry have research applications for detection or identification of the modified proteins. Versions that act as metabolic inhibitors of fucosyl transferase activity are of interest as research tools and as potential agents for clinical use (reviewed in Ref. 26). An issue is the need for inhibitors of specific fucosyltransferases such as POFUT2: for example, a phase I clinical trial of an inhibitory fucose analog in patients with advanced solid tumors indicated initial antitumor activity, but the trial had to be halted due to occurrence of thromboembolic events (27).

### Hydroxylation of Proline and Lysine Residues: Requirements in Functional Assembly of Collagens

**Figure 2.**
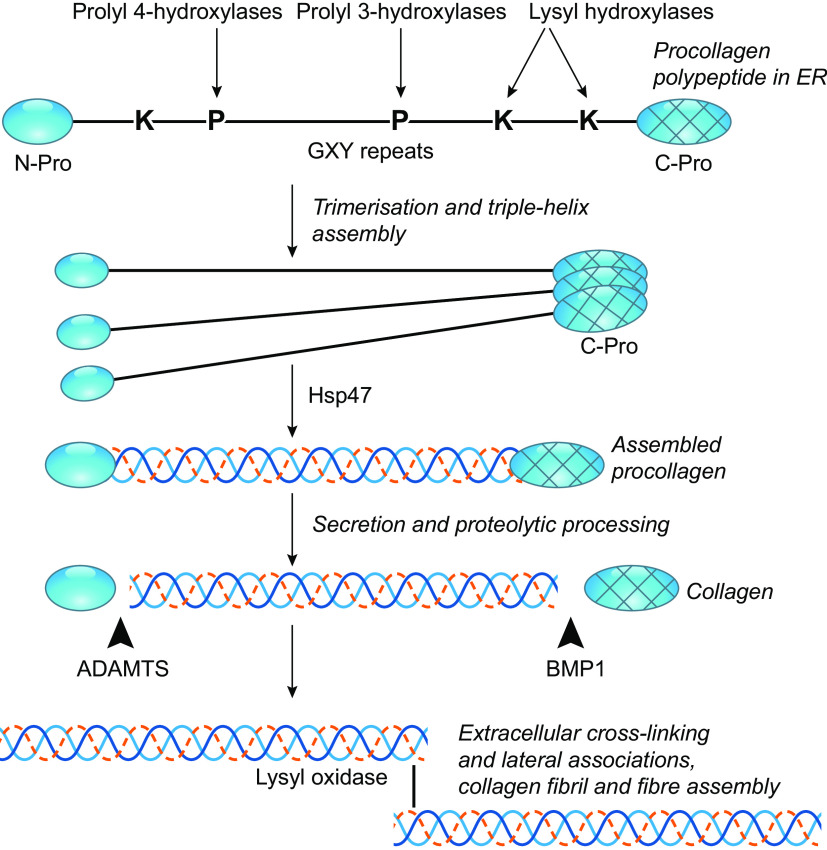
Role of proline and lysine hydroxylation PTMs in the maturation and assembly of fibrillar collagen molecules. The schematic presents major steps in the processing of fibrillar collagens, with the prolyl and lysyl hydroxylases and schematized target residues in bold. See text for details. Adapted from Fig. 2*B* of Ref. 29, and reproduced under author’s permitted use of Portland Press. ER, endoplasmic reticulum; PTMs, posttranslational modifications.

Fibrillar collagens are the most abundant components of ECM in connective tissues, where they are assembled into fibril and fiber structures that can reach up to 10 microns in diameter and many tens of microns in length (28). The complex intracellular and extracellular molecular mechanisms by which procollagen polypeptides are assembled into trimeric procollagen molecules, which are then secreted and processed for fibril assembly and bundling, have been the subject of intensive research over many years and are outlined in Fig. 2. Fibrillar procollagen polypeptides all contain extended central regions that consist of repeated GlyXY motifs, (where X and Y may be any amino acid but frequently include proline at the X position and the modified 4-hydroxyproline at the Y position). It is these extended GlyXY regions, which adopt a left-handed, α-helical like coil, that is responsible for the formation of the collagen triple helix as a right-handed super-helix. This process is initiated following chain registration of three procollagen polypeptides through covalent and noncovalent interchain interactions of their C-propeptide domains (28, 30). In this multistep trimer assembly process, the hydroxylation of proline residues (to 3-hydroxyproline or 4-hydroxyproline) and lysine residues (to 5-hydroxylysine) are PTMs of fundamental importance at the earliest steps (31; Fig. 2). The 4-hydroxyproline residues have essential roles in the stability of triple-helix assembly (32); initially thought to involve increased interchain hydrogen bonding, the activity appears to depend on stereoelectronic effects involving conformational alteration in the proline imino acid ring and a dominant role of hydroxyproline at the Y position of GlyXY motifs (33, 34). Reduced thermal stability is also associated with reduced collagen secretion or diminished fibril assembly (28). Functions of 3-hydroxyproline are less understood but are thought to include roles in the interactions of collagen molecules during fibril assembly, and possibly in the interactions of the fibrillar collagens with other fibril-associated ECM proteins (35). 3-hydroxyproline is also found in the basement-membrane forming collagen IV, which is a major substrate for prolyl hydroxylase 2 (36; Table 1).

**Table 1.** Proteins discussed in this review that are associated to human genetic diseases

Gene	Protein	LoF Molecular Phenotype	Human Genetic Disease	OMIM Ref.
*P3H1*	Prolyl 3-hydroxylase 1	Low 3-hydroxylation of Pro986 of COL1A1; excess lysyl hydroxylation and glycosylation of helical domain; delayed collagen folding	Osteogenesis imperfecta type VIII	610339
*P3H2*	Prolyl 3-hydroxylase 2	Loss of 3-hydroxyproline at sites on collagen I, II, and IV in the eye	High myopia and other eye defects; basement membrane nephropathy	610341
*P3H3*	Prolyl 3-hydroxylase 3	Under hydroxylation of Lys at collagen triple-helical domain cross-linking sites; reduced thermal stability of skin collagen; altered collagen fibril diameters	None known but some phenotypic similarities to Kyphoscoliotic Ehlers-Danlos syndrome	610342
*P4HA1*	Prolyl 4-hydroxylase subunit α 1	Reduced prolyl 4-hydroxylase activity; reduced collagen thermal stability; loss of basement membrane organization	Possible roles in congenital disease of connective tissue	176710
*P4HA2*	Prolyl 4-hydroxylase subunit α 2	Reduced 4-hydroxyproline content of collagens; minor reduction in thermal stability (collagen II)	Autosomal dominant myopia 25	600608
*P4HB/PDIA1*	Prolyl 4-hydroxylase subunit β	Mutations in a′ domain affect PDI activity; reduced oxidoreductase activity	Cole-Carpenter syndrome-1	176790
*PLOD1*	Lysine hydroxylase 1/Procollagen-lysine, 2-oxoglutarate 5-dioxygenase	Reduced lysyl hydroxylase activity; reduced hydroxylysine content of collagens; altered collagen fibril morphology	Kyphoscoliotic Ehlers-Danlos syndrome	153454
*PLOD2*	Lysine hydroxylase 2	Loss of hydroxylation of telopeptide lysines of collagen I; reduced trivalent collagen I cross linking	Bruck syndrome 2	601865
*PLOD3*	Lysine hydroxylase 3	Loss of glycosyltransferase activity on galactosyl hydroxylysine residues in collagens or other extracellular proteins with a collagenous domain; defective basement membrane structure	Bone fragility with contractures, arterial rupture, and deafness	603066
*B3GLCT*	β-1,3-glucosyltransferase	Reduced glucosyltransferase activity to O-fucosylated serine or threonine in specific motifs of TSR domains; reduced secretion of cognate TSR proteins	Peters-plus syndrome	610308
*FAM20C*	Family with sequence similarity 20, member C	Reduced phosphorylation of FAM20C substrates; reduced activity of chondroitin 4-O-sulfotransferase-1; reduced FAMC20 secretion	Raine syndrome	611061

See OMIM entries for further details. GGT, glucosyltransferase; LoF, loss of function; OMIM, Online Mendelian Inheritance in Man (https://www.ncbi.nlm.nih.gov/omim/); TSR, thrombospondin type 1 repeated domain.

The hydroxylation of proline or lysine residues is carried out by a suite of ER-resident enzymes that act only on the individual procollagen polypeptides (i.e., before triple-helix assembly). The extent of modification by these PTM is thus inherently linked with the rate of triple-helix assembly. 3-hydroxylation of proline residues is accomplished by prolyl-3-hydroxylases of differing collagen preference and tissue expression (37). Prolyl 3-hydroxylase 1 shows specificity for Pro4-HyProGly where the unmodified Pro is at the X position of a GlyXY motif (35), with a major site of modification in the collagen I alpha 1 (COL1A1) polypeptide being Pro986 (38). 4-hydroxylation of proline residues in the Y position is the most common form of hydroxyproline in collagens and is accomplished by several prolyl 4-hydroxylases in mammals, of which prolyl 4-hydroxylase A1 is the most ubiquitous (Fig. 2 and Table 1; 39). The relative frequency of the modifications is illustrated by a mass spectrometry study of ECM isolated from cultured human lung fibroblasts from patients with idiopathic pulmonary fibrosis (IPF), which analyzed effects of transforming growth factor beta (TGFβ) on hydroxylation and glycosylation of collagens. The analysis of collagen I A1 polypeptide (COL1A1) alone identified 109 sites containing 4-hydroxyproline at the “Y” position of the repeated GXY motif, along with 12 sites containing 3-hydroxyproline at the “X” position, of which 3-hydroxylation of Pro771 was strongly increased by TGFβ treatment (40). At the tissue level, collagen hydroxylation modifications undergo dynamic changes with physiological context; for example, as shown by a proteomic study of zebrafish hearts regenerating after ventricular amputation in which site-specific 3-hydroxyproline, lysine hydroxylation, and O-glycosylation of hydroxy-lysine were profiled over time across 23 collagen chains (41).

There are three lysyl hydroxylases in mammals that are responsible for 5-hydroxylation of lysine residues (Table 1). Hydroxylation sites in the fibrillar collagens include lysine residues within the telopeptides that flank each end of the triple-helical domain, as well as lysine residues within the triple-helical domain itself (Fig. 2). Telopeptide lysines are substrates for lysyl hydroxylase 2, whereas lysines within the central triple-helical domain are substrates of lysyl hydroxylases 1 and 3 (39). Within the triple-helical domain, only lysine residues in the Y position of the GlyXY motifs are substrates for hydroxylation. The telopeptide hydroxylysines are essential for cross linking of mature collagen molecules by lysyl oxidase; this step takes place in the later, extracellular steps of collagen fibril assembly (42; Fig. 2). Essential roles of hydroxylysine are indicated by the early embryonic lethal phenotypes of gene-knockout mice for lysyl hydroxylase 2 or 3 (43–45) and weakness, locomotion problems, and shortened lifespan in *plod1*-null mice (46).

Hydroxylysine residues may also undergo an additional O-glycosylation modification, in which either galactosyl hydroxylysine (a monosaccharide) or glucosylgalactosylhydroxylysine (a disaccharide) is substituted onto the 5-hydroxyl group. This pathway, also occurring in the ER, involves the action of glycosyl transferase 25 domain 1 or glycosyl transferase 25 domain 2 to add the first sugar group and lysyl hydroxylase 3 to then add glucose to form the disaccharide (47). This glucosyltransferase activity of lysyl hydroxylase 3 is independent of its lysyl hydroxylase (LH) activity (48). Whereas *plod3*-null mice die at E9.5 due to failure of basement membrane formation with associated apoptotic cell death (43, 44), mice expressing a *plod3* mutant lacking LH activity but with normal glucosyltransferase activity developed normally and were viable, albeit with reduced thickness of the lamina densa of the epidermal basement membrane at birth. These results indicated that the glucosyltransferase activity of lysyl hydroxylase 3 is essential for localization of collagen IV to basement membranes and the assembly of functional basement membranes during development (44).

The physiological importance of lysine and proline hydroxylation for the stability of collagen molecules, an essential requirement for their assembly into fibril structures, is further demonstrated by the human genetic disorders that result from loss-of-function mutations in several of the hydroxylase enzymes (Table 1). Altered activity of the hydroxylases is also associated with chronic pathophysiological conditions such as tissue fibrosis or cancer progression. For example, high expression of *P4HA1* and *P4HA2* is associated with reduced overall survival in human patients with breast cancer (49). In the same study, shRNA-silencing of either transcript in MDA-MB-231 human cancer cells inhibited xenograft tumor growth or spontaneous metastasis in immunocompromised mice. The secretion of collagen I was decreased in culture, and the capacity of cells to invade a cell-derived ECM was reduced. Similarly, in vivo, collagen associated with tumors, its hydroxyproline content, and the numbers of circulating tumor cells (a maker of effective invasive capacity) were all strongly reduced. Treatment of mice bearing control MDA-MB-231 xenograft tumors with the prolyl hydroxylase inhibitor, ethyl 3,4-dihydroxybenzoate, effectively reduced the collagen content of tumors and their metastasis to lung, thus suggesting prolyl hydroxylase inhibition as a new potential anticancer therapy (49). Subsequently, a number of prolyl hydroxylase inhibitors have been studied in vitro but issues related to toxicity, side effects, or specificity have limited their translation to the clinic. Efforts continue to identify new categories of inhibitors (50, 51).

Changes in the abundance of lysine hydroxylation of collagens also occur in fibrosis or cancer progression. Increased hydroxylation of telopeptide lysine residues due to increased activity of lysyl hydroxylase 2 resulted in increased lysyl oxidase-mediated collagen cross linking, reduced susceptibility of collagen to extracellular proteolysis, and mechanical stiffening of the ECM (52). These changes in ECM organization can support tumor expansion, tumor cell invasion, and metastasis due to positive effects on various forms of tumor cell migration. Excess and stiffened ECM also provides physical barriers to the access of therapeutic agents to the tumor cells (53–55). Although an inhibitor active on the three lysyl hydroxylases, minoxidil, has limited effects on collagen cross linking in vitro, there is considerable interest in developing specific inhibitors of lysyl hydroxylase 2 as antitumor or antifibrotic agents (42, 56).

### FAM20C Kinase: A Global Mediator in Phosphorylation of Extracellular Matrix Proteins

The biological activities of the family with sequence similarity 20 member C (FAM20C) kinase demonstrate the importance of the PTM of phosphorylation for the secretion of many ECM proteins. The FAM20 proteins (FAM20A, FAM20B, and FAM20C) were identified from cDNA and also characterized as transcripts expressed in hematopoietic cells. All FAM20 proteins contain a secretory signal peptide and are most related within their C-terminal regions, which correspond to the kinase domain; FAM20A and FAM20C also contain a disordered region near the N-terminus (57, 58). Mouse Famc20 (named at the time as Dentin matrix protein 4) showed calcium-binding activity and roles in odontoblast differentiation (59). In humans, mutations in *FAMC20* are causal for Raine syndrome, a lethal osteosclerotic bone dysplasia characterized by hardening and increased density of bone in which individuals die of respiratory failure (60), and are also associated with nonlethal osteosclerotic bone dysplasia phenotypes (61, 62; Table 1).

It has long been appreciated that many extracellular proteins, including ECM proteins, are serine/threonine phosphorylated, but the kinase involved remained uncertain until relatively recently. *FAM20C* is widely expressed in human tissues, and the protein was identified to be localized predominantly in the Golgi apparatus of cultured cells and function as a serine kinase that phosphorylates proteins destined for secretion on Ser-X-Glu/pSer peptide motifs (63). The first substrates identified included caseins and secreted proteins of bone cells with roles in biomineralization such as the small, integrin-binding ligand N-linked glycoproteins (SIBLINGS) and bone morphogenetic proteins (63–65). Expression of known Raine syndrome mutations of *FAM20C* in an osteosarcoma cell line showed that these mutations reduced FAM20C kinase activity and, in some cases, its secretion (66). An additional activity of FAM20C is to bind, phosphorylate and augment the activity of chondroitin 4-O-sulfotransferase-1 activity to promote 4-sulfation of chondroitin sulfate glycosaminoglycan chains. This binding activity is lost in four lethal Raine syndrome mutants of *FAM20C*, which all promoted biomineralization in an osteosarcoma cell culture model. Thus, perturbation of FAM20C interaction with chondroitin 4-O-sulfotransferase-1, as well as loss of kinase activity, may both contribute to the Raine syndrome bone dysplasia phenotypes (67). Conditional knockout of *fam20c* in mice was associated with softened bones resulting in rickets, and in serum, reduced phosphate concentration, and elevated fibroblast growth factor 23, supporting that FAM20C has a major role in osteoblast differentiation (68).

Overall, FAM20C has emerged not only as a key kinase for the phosphorylation of bone or dental matrix proteins but also as the central serine kinase that is responsible for phosphorylating most secreted phosphoproteins. More than 66% of the known phosphoproteins of human serum, plasma, or cerebrospinal fluid contain the FAM20C Ser-X-Glu/pSer substrate motif and a comparative quantitative proteomics study of conditioned media from wild-type and *FAM20C* knockout human cell lines (HepG2 and others) identified more than 100 extracellular proteins to undergo FAM20C-dependent serine phosphorylation, including fibronectin, laminin subunits, osteopontin, versican, and other ECM-associated proteins (69). Further studies have identified substrate proteins that reside within the secretory pathway (70). Although FAM20C contains a secretory signal peptide and can be identified extracellularly (71), it is considered that its kinase activity is enacted predominantly within the late secretory pathway (72). The wide substrate range of FAM20C is consistent with the evolutionary conservation of a *Fam20c* gene in invertebrate animals that lack mineralized bones or teeth (73).

Several biological modulators of FAMC20 kinase activity have been identified, although this remains an under-explored area. The related FAM20A (mutations in which are associated with amelogenesis imperfecta type 1 G; OMIM 611062) has been identified as a pseudo-kinase that forms a hetero-tetrameric complex with FAM20C, thus increasing the substrate-specific phosphorylation activity of FAM20C (74). FAM20A also promotes the extracellular localization of FAM20C (71). Sphingosine independently stimulates FAM20C kinase activity, especially in the presence of magnesium chloride (75). Kinase activity and secretion of FAM20C are also regulated by site-1 protease (S1P), which cleaves FAM20C at residue 92–93 to promote kinase activity and release of mature FAM20C for trafficking beyond the Golgi and eventual secretion (72).

Not unexpectedly, given the widespread expression of many FAM20C substrates in nonmineralized tissues, experiments with tissue-specific *fam20c*-knockout mice or *FAM20C*-depleted or knockout cells, have revealed additional functional roles to support cell and tissue functionality in nonmineralizing organs. As examples, cardiomyocyte-specific deletion of *fam20c* in mice increased their susceptibility to age-related or induced heart failure (76) and indeed several fam20c substrates of cardiomyocytes (such as histidine-rich Ca-binding protein, STIM1, or calsequestrin-2), have important roles in calcium homeostasis of the sarcoplasmic reticulum that are protective for failing hearts (76, 77). In contrast, *fam20c* knockout in the salivary glands of mice resulted in reduced numbers of acinar cells, alterations to organization of the ductal epithelium, and reduced production of saliva (78).

Other activities identified for FAM20C include promotion of the platelet-adhesive activity of von Willebrand Factor (79) or facilitation of lysosomal degradation of low-density lipoprotein receptor (80). The protein also has roles in ER homeostasis: through its phosphorylation of the luminal protein, sulfhydryl oxidase ER oxidoreductin 1α (Ero1α). In the Golgi apparatus, FAM20C contributes to cellular redox homeostasis by activating Ero1α to traffic to the ER to catalyze the formation of new disulfide bonds in newly synthesized proteins (70). FAM20C also functions in the ER stress response through phosphorylation of protein disulfide isomerase A1 (PDIA1, discussed in the next section); this PTM acts as a molecular switch to promote the chaperone activity of PDIA1 for protein refolding over its prominent oxidoreductase activity (81).

Given its central role for the delivery of ECM proteins to the extracellular milieu, FAM20C is of great interest as a potential therapeutic target. Research interest has centered on its emerging roles in tumor biology and relevance to the composition of ECM in the tumor microenvironment (TME). FAM20C has been associated with poor survival in glioblastoma (82) and was identified as an independent risk factor in low-grade gliomas (83). It also correlated with poor survival outcomes in bladder urothelial carcinoma and gastric adenocarcinoma (84). In breast cancer models, knockout of *FAM20C* in human MDA-MB-231 invasive breast cancer cells reduced cell attachment to culture surfaces and resulted in decreased cell migration in “scratch-wounds,” chemotaxis, and Matrigel invasion contexts (69). Complex roles of FAM20C activity in the TME are indicated by a mouse model of breast cancer metastasis to bone, in which deletion of *fam20C* from myeloid cells promoted osteoclast differentiation and bone resorption through increased osteopontin secretion. These results imply that normal expression of FAM20C by osteoclasts serves to produce a bone environment less favorable to bone metastasis of breast cancer cells. In contrast, expression of *FAM20C* in breast cancer cells acted as a driver of cell proliferation, tumor growth in vivo, and development of bone metastases in a mouse cardiac injection model (85). These opposing roles illustrate how clinical use of FAM20C inhibitors is likely to require cell-type-specific targeting. At present, development of such inhibitors is at an early preclinical stage. The FAM20C inhibitor, FL-1607, developed through in silico network analysis, molecular modeling, and molecular dynamics approaches, inhibited proliferation of MDA-MB-468 and MDA-MB-231 human breast cancer cell lines and increased apoptosis and inhibited migration of MDA-MB-468 cells (86). Similar inhibitory properties have been reported for a separate inhibitor, Fam20C inhibitor 3r (87).

## PROTEIN DISULFIDE ISOMERASES: CENTRAL ROLES IN DISULFIDE BOND FORMATION IN THE ENDOPLASMIC RETICULUM

As introduced with reference to the TSR domain, formation of disulfide bonds is a critical step in the initial folding, refolding, and final structure of newly synthesized polypeptides within the ER (88). This PTM is very relevant to ECM proteins, which typically contain many domains with intramolecular disulfide bonds or are frequently oligomerized through the formation of intersubunit disulfides (e.g., thrombospondins, fibronectin, and tenascins). Enzymes fundamental to disulfide bond assembly and isomerization in the ER are the protein disulfide isomerases (PDIs), of which 21 family members are encoded in the human genome (89, 90). The most-studied family member, PDIA1, is the most abundant enzyme within the ER. In addition to its disulfide oxidoreductase/isomerase activity, PDI also has molecular chaperone activities involved in ER-associated degradation of misfolded proteins (91). PDI activity is increased under ER stress and contributes to the unfolded protein response (89, 90). PDIA1 also constitutes the β subunit (P4HB) of prolyl 4-hydroxylases that functions in collagen biosynthesis. In this context, two PDIA1 molecules form a tetramer with two prolyl 4-hydroxylase α subunits (92).

**Figure 3.**
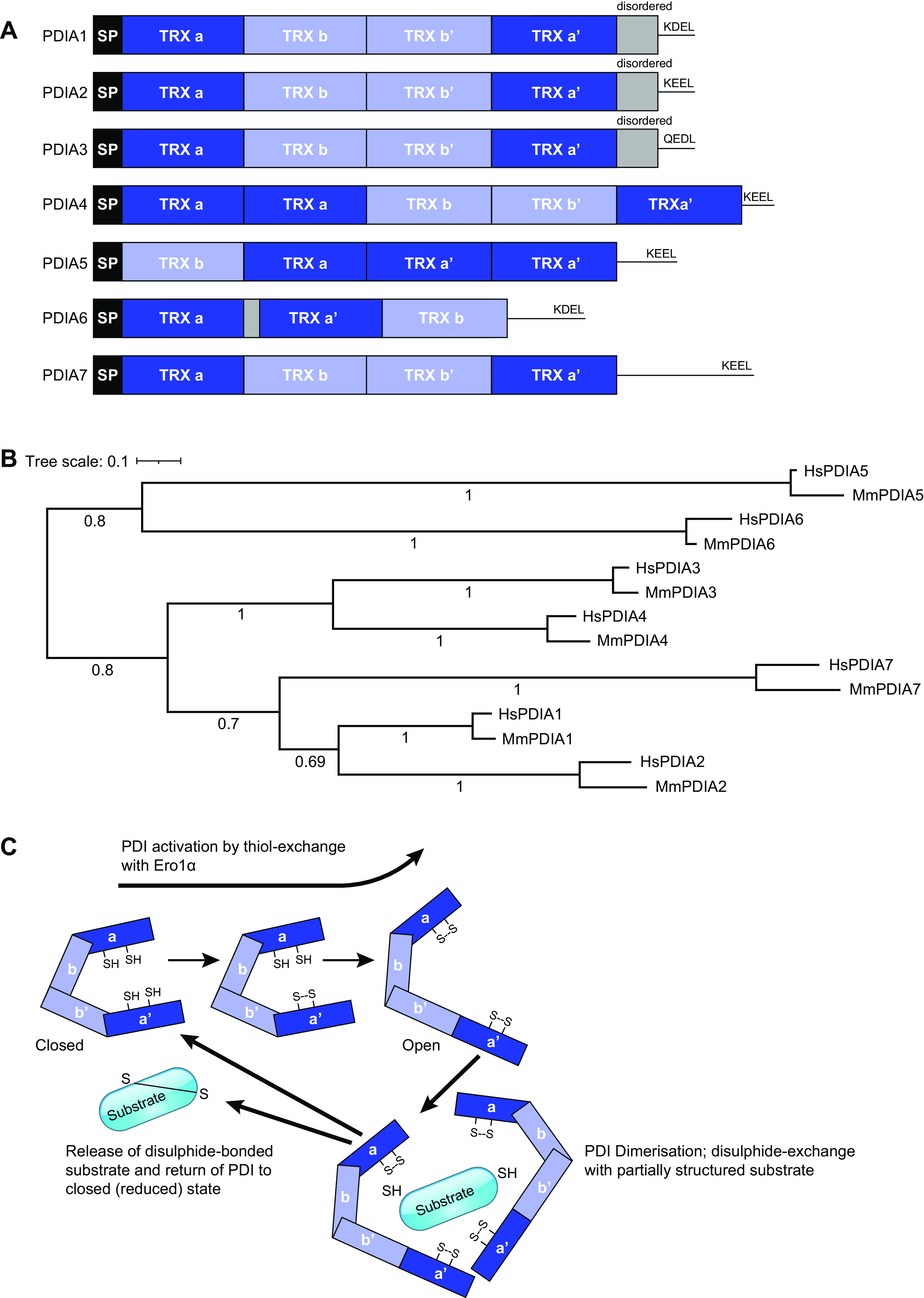
Classical PDIs of mammals. *A*: schematics of domain architecture, based on the human proteins. Cross-hatched regions indicate short, disordered regions, as identified by InterProScan 92.0 (58). SP = secretory signal peptide. *B*: protein sequence similarity relationships of the classical PDIs from human (Hs = *H. sapiens*) and mouse (Mm = *M. musculus*). Analysis was conducted at phylogeny.fr (93), with alignment of sequences by MUSCLE and tree construction in PhyML at default parameters and with 100 cycles of bootstrapping. The tree was visualized in iTOLv6.6; numbers refer to bootstrap values (94). *C*: schematic diagram of the redox mechanism of PDI. Oxidation of reactive thiols of the CysXXCys motifs in the a and a′ domains results in an “open” conformation of PDI which undergoes substrate-driven dimerization. Through thiol exchange reactions with the substrate protein, the substrate reaches its final, disulfide-bonded, folded conformation and is released for anterograde trafficking and PDI is returned to the reduced, closed state. PDI, protein disulfide isomerase.

At a molecular level, all PDIs are characterized by a secretory signal peptide and at least one thioredoxin-like domain. Many members contain a KDEL or related ER-retention motif at their C-terminus and some contain short-disordered regions. The thioredoxin domains of PDI proteins fall into two functional categories: the a-type domain, which contains a redox-active CysXXCys motif (with Gly-His common in the XX position), or the b-type domain, which lacks the CysXXCys motif and in general acts to tether substrate proteins through protein-protein interactions (89). I focus here on the classic PDIs, which comprise PDIA1 through PDIA7. These enzymes have multifunctional roles in disulfide bond formation, reduction, or isomerization within the ER and share commonalities of domain architecture, including multiple a-type and b-type thioredoxin domains. PDIA1 through PDIA3 and PDIA7 contains four thioredoxin-like domains, arranged a, b, b′, a′, whereas PDIA5 and PDIA6 contain a single b-type domain, and PDIA4 is a larger protein with an additional a-type domain (Fig. 3*A*). Despite the distinctions in domains, PDIA3 is most closely related to PDIA4 in terms of sequence similarity (Fig. 3*B*). PDIA1 is the only family member associated with a human genetic disease, Cole-Carpenter syndrome (Table 1); in addition, rare exonic variants of *PDIA1* and *PDIA3* have been identified in patients with amyotrophic lateral sclerosis that affect motor neuron connectivity (95). Gene-knockout of *pdia3* is early embryonic lethal in mice (96).

Oxidoreductase activity, as established from studies of PDIA1, involves the reactive thiol groups of cysteines within the CysXXCys motifs and disulfide exchange regulatory interactions between PDIA1 and the major ER oxidase, Ero1α. Ero1α binds to reduced PDIA1 and oxidizes it, resulting in stepwise formation of intramolecular disulfide bonds between the thiol groups of cysteines within the CysXXys motif of the a′ domain and then the a domain (97). This causes PDIA1 to undergo a conformational change from a “closed” to an open, more elongated state. Dimerization of oxidized PDIA1 in the presence of unfolded substrates forms an enlarged hydrophobic “cave” in which PDIA1 acts as an electron donor to substrates, resulting in their oxidative folding (90, 97). The scope of PDIA1 catalytic activity includes oxidation of Ero1α itself and of other PDI family members, linking these proteins into a redox regulatory network. Through the substrate oxidation reaction, PDIA1 is returned to a reduced state of its reactive thiol groups, to undergo further cycles of thiol-exchange [(98), reviewed by Wang et al. (99)]. Whereas PDIA1 interacts directly with substrates, which bind its b′ domain, PDIA3 has a distinct mechanism and interacts indirectly, via the ER lectin-type chaperones, calnexin and calreticulin, which bind to its b′ domain (90, 100). PDIA3 shows preference for monoglycosylated substrate proteins and identified substrates relevant to the ECM include collagen α(VI), laminin β and γ chains, lysyl oxidase homolog 2, and matrix metalloproteinase 9 (101–104).

## PDIs IN CANCER: RELEVANCE TO THE TUMOR MICROENVIRONMENT

Changes to ECM composition, organization, and mechanical properties are a hallmark of cancer and an important feature of the TME, which may facilitate migration, local invasion, and metastatic dissemination of tumor cells. ECM alterations also support plasticity of tumor cell phenotype, such as in epithelial-mesenchymal transition (105). These changes result from aberrant production of ECM proteins/ECM-associated proteins by tumor cells and also the intercellular communications by which tumor impact the cell phenotypes and ECM production/assembly by local nontransformed cells. Thus, the conversion of fibroblasts to cancer-associated fibroblast phenotypes, or the production of cytokines and ECM-degrading proteases by immune cells are also important drivers of alterations to the TME (106, 107). So-called neoepitope PTMs of tumor ECM have been associated with cancer progression and may serve as useful biomarkers (108, 109).

The availability of large numbers of sequenced cancer genomes has enabled the analysis of mutations at sites of PTM within ECM and matrisomal proteins. Of 2.3 million nonsilent mutations in 9,075 patients and 32 tumor types extracted from The Cancer Genome Atlas, 151,088 related to matrisomal proteins were identified and 1,811 (1.87%) of these affected sites of posttranslational modification (phosphorylation, acetylation, hydroxylation, N- and O-glycosylation, methylation, sumoylation, and ubiquitylation were studied; 110). The relative infrequency of mutations at PTM sites suggested that they are selected against, providing an indirect indication of the importance of PTMs for the functionality of matrisomal proteins within the TME.

Relevant to the PTM of disulfide bond formation, there is increasing evidence for altered expression of PDI family members in various forms of cancer. Table 2 summarizes evidence related to PDIA1, PDIA3, and PDIA6 in human breast cancer. Of these PDI family members, PDIA3 is of particular interest with regard to the TME, given its established glycoprotein substrate preference that includes glycoproteins of the ECM (102, 104). Although much of the evidence on breast cancer association of PDIA3 remains correlative, derived from transcriptomic or proteomic studies (Table 2), a number of studies have investigated functional roles of PDIA3 in breast cancer cell lines or in vivo rodent models. These studies have been aided by the existence of various PDI inhibitors, albeit that none at present are entirely specific. For example, the inhibitor 16F16 has a higher affinity for PDIA3 than for PDIA1 or PDIA4 (139), whereas the inhibitor PACMA-31 has greater selectivity for PDIA1 (140). Thus, use of inhibitors in combination with comparative studies of cells silenced or gene knocked-out for a specific PDI provides a way forward to investigate functions of individual family members.

**Table 2.** Summary of reported correlations/associations of PDIA3 and other classical PDI family members with human breast cancer cell functions and/or clinical samples

PDI Family Member	Correlation/Functionality in Human Breast Cancer Cells or Tumors			Reference
	Biological Sample	Major Expt. Methods	Results	
PDIA1/P4HB	IDC and adjacent normal breast tissue	Proteomics	PDI among a set of proteins increased in IDC sample	(111)
	Tissue samples of 9 breast cancers from male patients	2D IEF/NEPHGE and proteomics; immunoblot, IHC	PDIA1 among proteins increased in tumor vs. normal tissue	(112)
	Tumor (*n* = 69) or normal (*n* = 28) breast interstitial fluid; TMA	Proteomics; IHC; TMA	PDIA1 among the consistently greater than twofold upregulated proteins in tumor interstitial fluid; confirmed on TMA	(113)
	Samples of 11 primary breast cancers and matched axillary lymph node metastases	2-D-gel electrophoresis; Proteomics; IHC	PDI among the proteins consistently upregulated in lymph node metastasis vs. primary tumor	(114)
	MCF10A, MCF7, and MDA-MB-231 cells	Q3Rut, PACMA-31, and 16F16 inhibitors; assays of cell proliferation, attachment, migration, and transendothelial invasion	Cell-specific effects on proliferation, adhesion, migration, adhesion to endothelial monolayers, invasion and collagen gel contraction; alterations to free thiols of integrins; cell-surface PDIA1 detected	(115)
	MCF-7 and MDA-MB-231 cells	Bepristat 2a inhibition	Reduced cell adhesion to ECM proteins and to human microvascular endothelial cells; reduced transendothelial migration	(116)
	MCF-7 and MDA-MB-231 cells; breast cancer patient samples	siRNA-silencing	Cell-type specific effects on ROS, GSH, and ATP levels; reduced cell-surface HLA-G; correlation of low *PDIA3* and high *HLA-G* with increased OS of patients with stage 2 breast cancer	(117)
	Plasma of 51 nonmetastatic patients with breast cancer receiving neo-adjuvant chemotherapy	Plasma proteomics; clinical correlations	Elevated P4HB correlated with patients with pathological incomplete response to therapy, also with tumor stage; high expression correlated with decreased disease-free survival time	(118)
	MCF-7 and MDA-MB-231 cells	siRNA-silencing, RNASeq	Identification of up/down regulated genes after silencing, comparison of gene expression changes after IFNγ or etoposide treatments; relevance of pro-oncogenic gene sets and ER-status to OS correlations.	(119)
	T47D and MDA-MB-468 cells	Affinity purification of endogenous biotinylated proteins and proteomics	Identification of PDIA1 as a binding partner of a disulfide bond disrupting agent, an agent known to downregulate HER2 family proteins and kill breast cancer cells.	(120)
	MCF-7 and MDA-MB-231 cells	Proteomic ID of PDI family members in cancer cell lines; novel PDIA1/ PDIA3 inhibitors	Antiproliferative activity of inhibitors; reduced cell adhesion to collagen I	(121)
PDIA3	MCF-7, T47D, MDA-MB-231, and MCF-12A cells	siRNA-silencing; cell proliferation and cell cycle analysis	Reduced proliferation of T47D cells upon *PDIA3* silencing; cell-cycle arrest in G1 phase; minor increase in apoptosis	(122)
	Samples of 1 DCIS, 2 IDC and matched normal breast tissue	Mass spectrometry, proteomics, immunoblot	PDIA3 included in a signature of significantly upregulated proteins in DCIS and IDC relative to normal breast tissue	(123)
	MDA-MB-231 cells and BO2 bone metastatic subline	Proteomics; transcriptomics; primary and bone tumor analysis in nude mice; siRNA or shRNA silencing	PDIA3 included in a suite of proteins upregulated in BO2 cells and also predicted to network with multiple up- or downregulated proteins and cell adhesion proteins. Silencing of *PDIA3* in BO2 cells reduced bone metastatic growth in vivo	(124)
	MDA-MB-468 cells	siRNA silencing; EGF receptor location and functional parameters	Silencing of PDIA3 led to reduced EGF receptor phosphorylation and internalization	(125)
	Samples of 45 primary IDC and 9 normal contra-lateral samples	RT-PCR for *PDIA3* and *PDIA6*	PDIA3 expression significantly increased in carcinomas, in tumors with lymph node metastasis and with grade III	(126)
	Five paired samples of IDC and nontumor breast tissue. No neoadjuvant therapy	2-D-polyacrylamide gel electrophoresis; mass spectrometry	4.8-fold increase in PDIA3 protein in IDC samples (among top 10 elevated proteins)	(127)
	MDA-MB-231 cells	shRNA silencing; cellular assays	*PDIA3* silencing led to protection against etoposide-induced apoptosis, PERK activation, G2 cell cycle arrest and reduced cell proliferation	(128)
	3 IDC and 3 invasive lobular carcinoma tissue specimens	2-D-polyacrylamide gel electrophoresis; mass spectrometry	PDIA3 upregulated in IDC relative to invasive lobular carcinoma	(129)
	SUM159PT and MCF10DCIS.com cells	shRNA silencing of *PDIA1, PDIA3*; Mammosphere anchorage-independent growth	Increased expression of *PDIA3* and ECM genes in mammospheres vs. 2-D culture; mammosphere volume growth decreased by *PDIA1* or *PDIA3* silencing	(130)
	MCF-7, MDA-MB-231, and HCC1937 cells	PACMA-31 and 16F16 inhibitors; CM from WT- and *pdia3*-null mouse embryo fibroblasts (MEF); cell adhesion and migration assays	16F16 more active than PACMA-31 in decreasing cell attachment, spreading, F-actin and focal adhesion organization and cell migration; reduced cell-adhesive activity of ECM of HCC1937 cells treated with 16F16; decreased activity of CM of *pdia3*-null MEF to support breast cancer cell spreading and F-actin or migration	(131)
	Triple-negative breast cancer samples and public databases	Mouse model, cellular methods; analysis of single-cell transcriptomes of breast cancer and infiltrating immune cells	PDIA3 present on the surface of tumor-associated macrophages from TNBC; High expression of *PDIA3* in macrophages of M2 phenotypes correlated with poor prognosis and immune suppression markers	(132)
	MDA-MB-231 cells	Inhibitor dihydrotanshinone I; Affinity chromatography and mass spectrometry; biochemical and cell-based assays	Dihydrotanshinone I identified as a new binding partner and inhibitor of PDIA3 redox activity; in cells, reduced viability, decreased PDIA3 protein, also increased ER stress and apoptosis	(133)
	MDA-MB-231 cells	16F16 inhibition; TMT-proteomics on CM; in. silico data analysis	16F16 treatment resulted in 80 proteins of reduced abundance in CM, including many networked ECM proteins; high expression of these correlated with reduced DMFS of basal-type patients with breast cancer	(134)
PDIA6	MCF-7 and MDA-MB-436 cells; breast cancer TMA	PDIA6 over-expression; phosphorylation status of ErbB2; activity state of ErbB2 pathway proteins; TMA	Increased migration or invasion and increased active ErbB2 in PDIA6-over-expressing cells; elevated PDIA6 in invasive ductal carcinomas (IDC)	(135)
	Samples of 45 primary IDC and 9 normal contra-lateral samples	RT-PCR for *PDIA3* and *PDIA6*	*PDIA6* transcript significantly elevated in subgroups with lymph node metastasis and significantly reduced with hormone-receptor negative status	(126)
	Five paired samples of IDC and nontumor breast tissue. No neoadjuvant therapy	2-D-polyacrylamide gel electrophoresis; mass spectrometry	5.2-fold increase in PDIA6 protein in IDC samples (among top 10 elevated proteins)	(127)
	3 IDC and 3 invasive lobular carcinoma tissue specimens	2-D-polyacrylamide gel electrophoresis; mass spectrometry	PDIA6 upregulated in IDC relative to invasive lobular carcinoma	(129)
	BT20, T47D, and MDA-MB-231 cells	Flow cytometry, immunoblotting	PDIA6 detected on the surface of breast cancer cell lines and in cell lysates	(136)
	HCC70 (triple-negative) and SKBR3 (HER2-positive) cells; breast cancer patients’ sera	siRNA screen for decreased breast cancer cell survival and increased apoptosis; mouse models; auto-antibody detection in patient sera	*PDIA6* silencing decreased cell survival and increased apoptosis; PDIA6 vaccination of mouse model decreased tumor growth by 43%; autoantibodies to PDIA6 elevated in sera from DCIS and invasive patients with breast cancer compared with controls, also in fibroadenoma condition	(137)
	MCF-7, T47D, MDA-MB-231 cells	Tamoxifen treatment of cells; isolation of aggresomes; mass spectrometry	Proteins associated with unfolded protein response, including PDIA6, incorporated into aggresomes in antiestrogen sensitive, but not antiestrogen-resistant cells	(138)

Samples from female patients unless otherwise stated. Based on publications listed in PubMed; breast cancer literature not identified for PDIA2, PDIA4, PDIA5, or PDIA7. ATP, adenosine 5′-triphosphate; CM, conditioned medium; DCIS, ductal carcinoma in situ; DMFS, distant metastasis-free survival; ECM, extracellular matrix; EGF, epidermal growth factor; GSH, glutathione; IDC, invasive ductal carcinoma; IHC, immunohistochemistry; OS, overall survival; PERK, protein kinase RNA-like endoplasmic reticulum kinase; ROS, reactive oxygen species; RT-PCR, real-time polymerase chain reaction; TMA, tissue microarray; 2-D, two-dimensional.

### Effects of PDIA3 Loss-of-Function in Breast Cancer Cells or Fibroblasts

Multiple studies have reported inhibition of cell proliferation and/or cell cycle progression in breast cancer cell lines upon pharmacological PDI-inhibition or *PDIA3* silencing (121, 122, 128, 133). In one study, PACMA-31 had stronger effects than 16F16 in inhibiting proliferation of MCF-7 or MDA-MB-231 cells (115). Depending on the cell lines and/or experimental design, PDIA3 inhibition led either to increased apoptosis (122, 133) or protected against apoptosis in response to a chemotherapeutic agent (128; Table 2).

**Figure 4.**
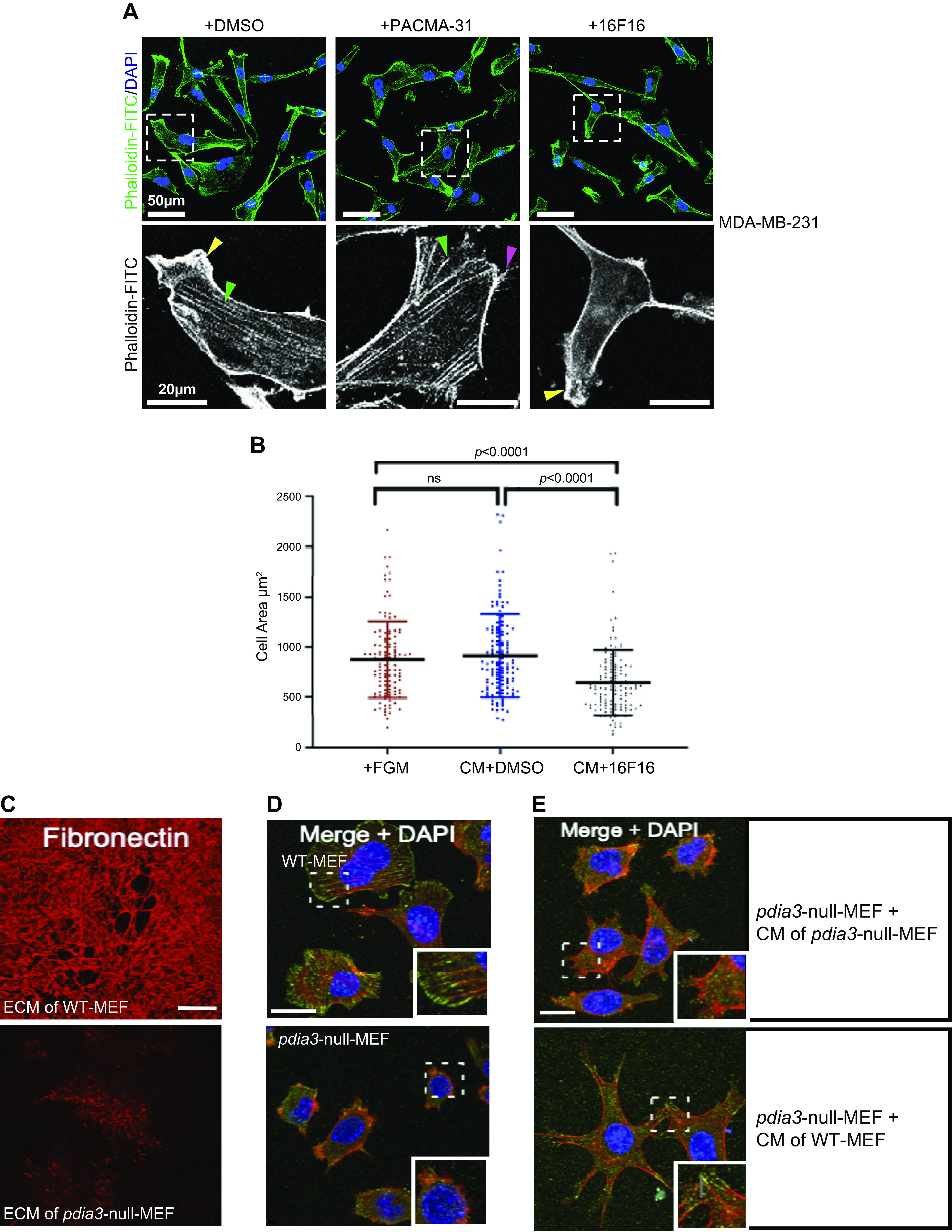
Effects of loss of PDIA3 activity on cell adhesion, fibronectin deposition, and functional properties of conditioned medium. *A*: comparison of effects of PACMA-31 (2.5 µM) or 16F16 (5 µM) or DMSO as solvent control on spreading and F-actin organization in MDA-MB-231 human breast cancer cells. Cells were plated in serum-free media for 2 h, then fixed and stained with FITC-phalloidin. Yellow arrows: lamellipodia; green: stress fibers; and purple: cell protrusions. Boxed areas are shown enlarged in the lower row. Reproduced from Ref. 131, published by Portland Press, and licensed open access under CC-BY 4.0. *B*: effect of 16F16 treatment on cell-adhesive properties of MDA-MB-231 conditioned medium (CM). Serum-free CM was prepared over 48 h, filtered, and incubated with newly plated MDA-MB-231 cells. After 4 h, adherent cells were fixed, stained with phalloidin-Atto 565, and cell areas measured in ImageJ. Each datapoint is a single cell, bars show mean, and SD reproduced from Ref. 134, published by American Physiological Society and licensed open access under CC-BY 4.0. *C*: lack of fibronectin deposition to ECM by *pdia3*-null MEF. WT-MEF or *pdia*3-null MEF were plated for 48 h, ECM isolated as described (141), and fibronectin visualized by indirect immunofluorescence. *D*: *pdia3*-null MEF are impaired for cell spreading and focal adhesion assembly in serum-free media. Cells were plated for 2 h, fixed, and costained with TRITC-phalloidin, antibody to vinculin, and DAPI; boxed areas also shown as enlargements. *E*: spreading and focal adhesion assembly by *pdia3*-null MEF is stimulated by exposure to CM of WT-MEF. CMs were prepared as in *B* and incubated with newly plated *pdia3*-null MEF for 2 h, and cells then fixed and costained with TRITC-phalloidin, antibody to vinculin and DAPI; boxed areas also shown as enlargements. Scale bars = 20 µm. *C–E*: reproduced from Ref. 142, published by American Physiological Society and licensed open access under CC-BY 4.0. ECM, extracellular matrix; TRITC, tetramethylrhodamine isothiocyanate; WT-MEF, wild-type mouse embryo fibroblasts.

In studies pertaining to cell adhesion and migration, pharmacological inhibition by 16F16 or PACMA-31 reduced breast cancer cell attachment to collagen I or vitronectin-coated surfaces but did not significantly reduce migration into “scratch wounds” on these substrata. MDA-MB-231 cells also attach to and transmigrate through endothelial cell monolayers; these activities were both inhibited by preincubation with PACMA-31 or 16F16 (115). These studies, which used relatively high concentrations of each inhibitor, implicated both PDIA1 and PDIA3 in control of cell interactions of breast cancer cells. A separate analysis, in which inhibitors were titrated individually for each cell line examined, reported that 16F16 inhibited spreading of breast cancer cell lines on glass or tissue-culture surfaces to a much greater extent than did PACMA-31 (131). The altered cell morphology correlated with decreased organization of focal adhesions and reduced F-actin-containing cell surface protrusions and stress fibers (all markers of effective cell adhesion), as shown for MDA-MB-231 cells in Fig. 4*A.* Not surprisingly, given these changes, 16F16 also inhibited cell migration in a quantified “scratch wound” model and proved more effective than PACMA-31. Decellularized ECM produced by basal-phenotype HCC1937 cells treated with 16F16 had reduced activity to support attachment and spreading of newly plated “naïve” HCC1937 cells than the ECM produced by control HCC1937 cells (131). Overall, the altered properties of ECM produced by PDIA3-inhibited cells suggested that loss of PDIA3 activity has a major effect on the secretion/extracellular interactions of ECM proteins. This was examined further by comparing initial cell adhesion of MDA-MB-231 cells plated under serum-free conditions either with fresh media or conditioned medium (CM) prepared from MDA-MB-231 cells treated either with 16F16 or a solvent control. CM from 16F16-treated cells had significantly reduced activity to support cell spreading (134; Fig. 4*B*).

Roles of PDIA3 have also been studied in nontransformed fibroblasts, where elevated expression or inhibition of PDIA3 increased or decreased, respectively, the production or incorporation of fibronectin into cell-derived ECM (142, 143). To investigate further how loss of PDIA3 activity affects deposition of ECM, localization of fibronectin was examined in cell-derived ECM produced either by wild-type mouse embryo fibroblasts (WT-MEF) or *pdia3*-null MEF. Fibronectin deposition and formation of a fibrillar network were severely perturbed in the *pdia3*-null MEF (Fig. 4*C*). Comparison of cell adhesion also showed that *pdia3*-null MEF had reduced attachment and spreading compared with WT-MEF under matched, short-term experimental conditions (Fig. 4*D*). Spreading of *pdia3*-null cells could be stimulated by plating in serum-free CM from WT-MEF, but not by exposure to CM from *pdia3*-null cells, providing further evidence of a key role of PDIA3 for production of secreted, proadhesive proteins (142; Fig. 4*E*).

These studies paved the way for identification of PDIA3-dependent, secreted proteins of WT-MEF by use of comparative quantitative tandem-mass tag proteomics on CM from WT-MEF and *pdia3*-null MEF. Relative to WT-CM, 21 extracellular proteins were greater than or equal to twofold decreased in and 7 proteins were greater than or equal to twofold increased in CM from *pdia3*-null MEF. The set of proteins was strongly enriched for Gene Ontology terms of “ECM” and “matrisome” and also enriched for the Gene Signature term “Epithelial-mesenchymal transition,” thus clearly demonstrating a functional importance of PDIA3 for extracellular production of a wide range of ECM and ECM-associated proteins. Of the new candidate PDIA3 substrates, cell communication network 2 (CCN2) was represented in all the Gene Ontology terms and also highly networked in protein-protein interaction maps. In further functional experiments involving either CCN2-immunodepletion or add-back of recombinant CCN2, CCN2 was identified to have a crucial role in the cell adhesion-promoting activity of CM from WT-MEF (142).

**Figure 5.**
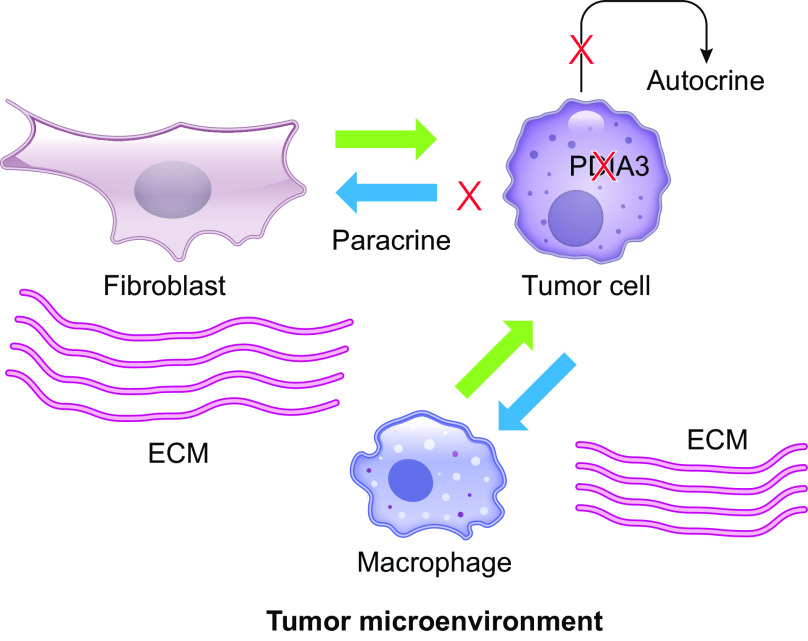
Schematic of breast cancer cell-host cell interactions in the tumor microenvironment and the role of paracrine and autocrine interactions in determining ECM composition and cell phenotypes. “X” indicates how loss of PDIA3 activity in tumor cells would inhibit autocrine and paracrine secretions, potentially leading to renormalization of the TME and decreasing the invasive potential of tumor cells. ECM, extracellular matrix; TME, tumor microenvironment.

Quantitative tandem-mass tag proteomics methodology was also used to identify proteins in the CM of MDA-MB-231 breast cancer cells that depend on PDIA3 activity, by comparison of proteins in the CM of control or 16F16-inhibited cells. In this analysis, 80 proteins were identified as greater than or equal to twofold decreased with *q* value of 0.05 or less over four independent experiments (no proteins were greater than twofold increased). These proteins included intracellular as well as extracellular proteins. Many of the intracellular proteins are known components of exosomes: indeed, since the method used did not exclude exosomes <200 nm from the CM, these proteins could have been captured as exosomal contents in the analysis. The set of extracellular proteins was strongly associated with Gene Ontology terms of “Cell adhesion,” “ECM” and related terms and included known PDIA3 substrates. Further experiments will be needed to discern which proteins are direct PDIA3 substrates and which may be “passengers,” i.e., proteins normally cotrafficked by binding to PDIA3 substrate proteins. All extracellular proteins except the protease SERPINE1 included disulfide bonds or disulfide-bonded domains, and the majority of these domains corresponded to β-hairpin folds, with the knottin fold family being the most frequently represented domain category (134). These results support prior identification of EGF domains in several PDIA3 substrates (102). *PDIA3* over-expression in breast cancer correlates with poor clinical outcomes (Table 2), and our analysis of public data sets in the GOBO database showed that high expression of a gene signature corresponding to the extracellular protein set correlated with reduced distant metastasis-free survival of patients with basal-subtype breast cancer (134). Overall, these findings suggest the concept that PDIA3 inhibition in tumor cells and/or local fibroblasts could be a relevant strategy to modify ECM properties and intercellular communications between tumor cells and local normal cell types, thereby driving “renormalization” of the TME to inhibit migratory and metastatic properties of invasive tumor cells (Fig. 5).

### Activities of PDIA3 in In Vivo Models of Breast Cancer Metastasis

To date, in vivo studies of PDIA3 in breast cancer have mostly explored its roles in metastasis, the major cause of mortality of patients with breast cancer. Despite improvements in breast cancer screening and early treatment over the last 40 years, 5-year survival for patients diagnosed with stage IV (metastatic) breast cancer is around 25% (144). Breast cancers most frequently metastasize to lung, liver, brain, or bone, with bone metastasis being the most common site of secondary tumors and often associated with reduced survival. A comparative study of the proteomes of MDA-MB-231 cells and a highly bone-metastatic subline, BO2, identified PDIA3 as a highly networked protein among the proteins of increased abundance in BO2 cells. Silencing of *PDIA3* in BO2 cells by shRNA reduced the ability of the cells to form bone metastases after injection in immunocompromised mice, with both the size and number of metastatic nodules being decreased (124).

The models discussed so far consider the ER as the main site of function of PDIA3, in accord with its location in nontransformed cells. However, extracellular PDIA3 has been reported, (e.g., Ref. 143), and there is evidence that cancer cells may traffic PDIA3 extracellularly to invadosomes (F-actin structures prominent on the basal surface of certain cancer cells, macrophages, or osteocytes where matrix-degrading proteases are concentrated) to participate directly in the susceptibility of ECM to degradation (145). In this process, PDIA3 was trafficked in complex with O-glycosylated calnexin. Once enriched in invadosomes, PDIA3-mediated reduction of disulfide bonds within the ECM rendered ECM collagens more accessible to proteolytic degradation by matrix metalloproteinases. This degradative activity could be reduced by knockdown of either calnexin or PDIA3, and the requirement for PDIA3 was related to its activity in reduction of disulfide bonds. In vivo, this process was examined in relation to breast cancer metastasis in an immunocompromised mouse xenograft model. MDA-MB-231 cells with high levels of cell-surface calnexin/PDIA3 had a greater capacity to form lung metastases and, as demonstrated by antibody blockade, the number of metastases depended on the availability of calnexin on cell surfaces (145). Thus, in establishing the mechanism of action of PDIA3 inhibitors in cancer cells, the possible localization of PDIA3 to different cell compartments or redox environments would need to be considered.

## LOOKING TO THE FUTURE

This review discusses selected examples to showcase the many ways in which PTMs of ECM proteins are essential for successful anterograde trafficking and secretion, or contribute as mediators or moderators of ECM interactions and functionality in the extracellular milieu. In the cellular context, proteins that add PTMs do not act alone but are associated in protein complexes or are coordinated through feed-forward or feed-back pathways. This complicates the functional consequences of inhibiting a single protein or category of proteins within the network, nevertheless the concept of manipulating PTM-enzymes to achieve quantitative or qualitative alterations to ECM production has appeal. Many ECM proteins, especially collagens, are very stable as well as very abundant in tissues, which puts limitations on the efficacy of using knockdown methods to reduce the levels of individual proteins. Use of small molecule inhibitors to block interactions between ECM proteins also poses challenges due to the abundance of the proteins and relatively low affinity of their natural interactions, although great progress is being made as specific binding motifs on fibrillar collagens are mapped (146). Instead, pharmacological inhibition of PTM-addition enzymes such as POFUT2 or PDIA3 offers the possibility to affect multiple ECM proteins with a single agent and to leverage coordinated changes in ECM quantity and/or quality, thereby altering the physiological properties of the microenvironment.

Indeed, as outlined, small molecule inhibitors of various PTM-enzymes have shown potential in preclinical research in cell or animal models relevant to fibrosis or cancer progression. To date, the cancer studies have relied mostly on xenografts or injections of human tumor cells in immunocompromised mice. Although these studies are useful pilots for proof of concept, they do not fully recapitulate the complex evolution and multiplicity of local host-tumor interactions associated with native tumor progression. Use of more sophisticated systems, such as tumor spheroids in vitro or mouse genetic models of cancer, would give a clearer view on, for example, the relative effects of PDIA3 inhibition on primary or metastatic tumors or effects on the ECM and TME over time. Research and development to identify inhibitors with suitable properties for clinical trials is ongoing. The available inhibitors of PDIs include some with substrate preferences between family members, or PDIA1 specificity, (139, 147), but an inhibitor fully specific for PDIA3 is yet to be developed. This would be a key tool for further investigations of the roles of PDIA3 in tumor progression and metastasis.

Another important translational area, in which it may be possible to make faster progress with applications for inhibitors of PTM-enzymes, is the production of so-called secretomes for cell culture, tissue engineering, or other translational purposes. Secretomes are prepared by the collection of serum-free CM from cells, organoids, or tissue explants and the composition reflects the totality of extracellular products of the cells. The material is often highly enriched in ECM proteins (148, 149) as well as other bioactive cell products such as cytokines, lipids, metabolites, or nanoscale membrane-bound particles. Secretomes have widespread research applications, such as the use of mesenchymal stem cell secretomes in regenerative therapies (150), or investigations of cancer stem cell secretomes for new anticancer therapies (151). Advantages of secretomes include the ability to readily work with a great variety of human primary cell types and also that these are acellular materials that can be applied allogenically in vivo (149). These properties have triggered much interest in the development of secretome materials for clinical purposes. Indeed, a search of the U.S. National Library of Medicine database of global clinical trials (clinicaltrials.gov) shows around 40 registered studies involving the use of secretomes or CM. It is clear that inhibition of PDIA3 leads to alterations in the protein profile and functional activities of CM in vitro (131, 134, 142). Thus, manipulation of PDIA3 or other PTM-enzymes in cells used for secretome production could be of value in basic and applied research, to change the properties of a secretome, or to research the basis of its functional properties.

## DISCLOSURES

No conflicts of interest, financial or otherwise, are declared by the authors.

## AUTHOR CONTRIBUTIONS

J.C.A. analyzed data; prepared figures; drafted manuscript; edited and revised manuscript; approved final version of manuscript.
